# Amphetamine-type stimulants use and socio-economic factors associated with hepatitis C antibody positivity among border drug users in South of China

**DOI:** 10.3389/fpubh.2022.998768

**Published:** 2022-10-25

**Authors:** Jing Li, Minyue Li, Yunjia Zhang, Jiashuang Li, Yinzhou Zhao, Rong Lu, Jing You

**Affiliations:** ^1^School of Public Health, Kunming Medical University, Kunming, China; ^2^Department of Geriatric Gastroenterology, The First Affiliated Hospital of Kunming Medical University, Kunming, China; ^3^Yunnan University Secondary School, Kunming, China

**Keywords:** amphetamine-type stimulants, hepatitis C virus, ATS, socio-economic factors, border area

## Abstract

**Background:**

Amphetamine-type stimulants (ATS) use has become popular in China. This study explored ATS use status and related risk factors of hepatitis C virus (HCV) infection among ATS users in Jinghong City, Xishuangbanna Prefecture, Yunnan Province, China.

**Methods:**

A cross-sectional study was conducted by questionnaires from January to July 2021 in border area in Yunnan. Respondent driving sampling and consecutive sampling was carried out among border drug users, and blood samples were tested for HCV antibodies. HCV infection and related risk factors among ATS users were measured. Descriptive, univariate and multivariate analysis were conducted separately by Software SPSS 26.0.

**Results:**

The ATS users accounted for 85.82% (345/402) among drug users, while anti-HCV antibody prevalence was 6.38% (22/345) among ATS users. The combined use of other types of drugs (*OR* = 7.29, *95%CI*: 1.982–26.81, *P* = 0.003), injection drug use (*OR* = 6.823, *95%CI*: 1.898–24.525, *P* = 0.003), average monthly income (*OR* = 4.825, *95%CI*: 1.325–17.566, *P* = 0.017) might increase the risk of HCV infection among ATS users. ATS users with high school or above had higher HCV infection rates than those with primary school or below (*OR* = 5.718, *95%CI*: 1.172–27.908, *P* = 0.031).

**Conclusion:**

Taken together, among drug users using ATS in Jinghong City, Xishuangbanna Autonomous Prefecture, Yunnan Province, combined use of multiple drugs and intravenous drug use was the high risk factor for HCV infection. Therefore, corresponding education and intervention measures should be taken.

## Introduction

Approximately 27 million people worldwide used amphetamine-type stimulants (ATS) in the World Drug Report 2020, while methamphetamine and amphetamine are now the world 's main forms of amphetamine-type stimulants available and the most widely consumed (1, 2), quickly replacing heroin as the most common drug in the country (3, 4). Compared to traditional drugs such as heroin and cannabis, ATS is one new type of drug. When ATS users are mixed with other types of drugs, they are more likely to have intravenous injections and high-risk sex behaviors. Research (5) predicted that new drugs such as ATS would likely replace heroin as a mainstream drug in China in the 21st century.

As a highly addictive stimulant, ATS could stimulate the secretion of dopamine in the human body, which possibly made people feel abnormally excited and euphoric, and increased people's sexual desire (6). People using methamphetamine had the high probability of non-lethal risks, mainly including long-term use of psychiatric symptoms such as paranoia (strong suspicion), auditory or visual hallucinations (7, 8) and violence and aggressive behavior (7), sexually transmitted diseases (9) and blood-borne infections (7). The main pathogens of blood-borne infections included human immunodeficiency virus, hepatitis B virus, and hepatitis C virus (HCV) usually associated with intravenous drug use and blood transfusion. Therefore, people who used drugs, especially those who injected drugs, became high-risk groups of HCV infection (10). Approximately 20% of people develop cirrhosis within 20 years of HCV infection, along with an increased risk of hepatocellular carcinoma and decompensated liver disease (11, 12).

As the number of ATS users increased, the risk of HCV infection associated with ATS possibly increased simultaneously. Gahrton et al. (13) reported that HCV-infected amphetamine users had higher all-cause mortality compared to the general population. However, there were few investigations on HCV infection and its correlation among ATS users especially in the border areas. Thus, we hypothesized that there were associations between HCV infection and drug abuse especially multiple drug uses among ATS users. This study aimed at description of HCV infection prevalence among ATS users in the border area, and exploration of risk factors associated with HCV infection for early prevention and control strategies among ATS used populations.

## Methods

### Setting and participants

The study was a cross-sectional study from January to July 2021 in Jinghong City, Xishuangbanna Autonomous Prefecture, Yunnan Province. There were two time periods for data collection.

From January to February 2021, 261 people were recruited at the center for disease control and prevention, methadone clinics and detention center (places awaiting referrals to drug rehabilitation centers); From May to July 2021, 145 people were recruited at the detention center again. Finally, there were a total of 406 target population by respondent driving sampling and consecutive sampling. Inclusion criteria for drug users were: ① people who had ever used or injected drugs; ② at least 18 years old. All respondents were aware of the purpose of this study and signed the informed consent form related to this study. The entire research procedure was designed to protect the privacy of the respondents and to participate anonymously and voluntarily. No names and personal identifiers were used in the data collection. Ethical approval was obtained through Ethics Committee of Kunming Medical University (KMMU2022MEC017). All participants gave written informed consent in accordance with the Declaration of Helsinki.

### Data collection and processing

The study mainly used self-made questionnaires, and the contents of the questionnaires had been discussed and modified in many ways, including sociodemographic data, personal life history, and use of illegal addictive drugs. This survey was conducted with respondent driven sampling and consecutive sampling methods. At the beginning of the study, the local center for disease control and prevention medical workers provided three respondents (seeds), and each seed was issued three referral cards (the detailed location and simple map of the investigation office were marked on the referral card). When the respondents completed the questionnaire and took blood samples, they would be rewarded 80 yuan. At the same time, each successful referral would increase the reward of 20 yuan per person. In the later referral process, due to the impact of the COVID-19 epidemic and the possibility of fear and vigilance among the respondents, fewer respondents were referred, so we actively recruited drug addicts in methadone clinics, detention center and other places. At the end of the investigation, a total of 406 respondents were recruited. Because four of them did not meet the study criteria, 402 were actually included in the study, of which 345 were using ATS drugs.

### Measures and variables

This research mainly adopted the form of structured questionnaire, and collected 3 ml serum samples of the respondents. At the same time, in accordance with the requirements of the “Technical Specifications for Laboratory Detection of Hepatitis C Virus”, the Elisa kit was used to detect the serum anti-HCV level of the researcher. The independent variable was whether anti-HCV reactive test (no = 0, yes = 1) and dependent variables included age group ( ≤ 25 = 1, 26–45 = 2, ≥46 = 3), education level (primary school and below = 1, junior high school = 2, high school and above = 3), marital status (unmarried/separated/divorced/widowed = 0, unmarried cohabitation/married = 1), average monthly income (< 4,000 Yuan = 1, 4,000–8,000 Yuan = 2, ≥8,000 Yuan = 3), nationality (Dai people = 1; Han nationality = 2; other = 3), gender (Female = 0, Male = 1), whether injecting drugs (no = 0, yes = 1), whether condoms were used (spouse/constant sexual partner, temporary partners; never used = 0, not always used = 1; used every time = 2; declined to answer = 3), whether smoking (no = 0; yes = 1), and whether to drink (no = 0; yes = 1).

### Statistical analysis

The questionnaire contents were input as statistical data using Epi data software, and SPSS 26.0 was used for statistical analysis of the obtained data. Basic descriptive data were represented by examples and constituent ratios; measurement data that conform to normal distribution were represented by mean ± standard deviation; measurement data that did not conform to normal distribution were represented by interquartile range, and nonparametric test analysis was used; enumeration data were represented by Chi-square test and was used to conduct one-way ANOVA; multivariate logistic regression analysis was used to analyze the correlation between each factor and HCV infection. *P* < 0.05 was considered statistically significant.

## Result

### Basic sociological characteristics of drug addicts

A total of 402 drug addicts were investigated this time, including 396 males, accounting for 98.51%, and six females, accounting for 1.49%, aged 18–72 years old, with an average age of (33.68 ± 9.8) years old, and most of them were Dai (41.79%). The education level was mostly concentrated in primary school and below (50.75%). 71.64% were unmarried, separated, divorced or windowed. 78 target population were intravenous drug users, accounting for 19.40%. A total of 85.82% (345/402) had used ATS, while 14.18% (57/402) had never used ATS. A larger proportion of the population drank (96.77%) or smoked (91.54%). See Table 1 for details.

**Table 1:** Demographic characteristics among drug users (*N* = 402).

**Variable**	***N* (%)**	**Variable**	***N* (%)/*M*(IQR)**
Age group		Whether condoms are used (spouse/constant sexual partner)	
≤ 25	78 (19.40)	Never used	123 (30.60)
26–45	270 (67.16)	Not always used	81 (20.15)
≥46	54 (13.43)	Used every time	53 (13.18)
		Declined to answer	145 (36.07)
Education level		Whether condoms are used (temporary partners)	
Primary school and below	204 (50.75)	Never used	51 (12.69)
Junior high school	151 (37.56)	Not always used	40 (9.95)
High school and above	47 (11.69)	Used every time	66 (16.42)
		Declined to answer	245 (60.95)
Marital status		Number of sexual partners	31 (27.00–39.00)
Unmarried/separated/divorced/widowed	288 (71.64)	Whether injecting drugs	
Unmarried cohabitation/married	114 (28.36)	No	324 (80.60)
Average monthly income		Yes	78 (19.40)
< 4,000 yuan	241 (59.95)		
4,000–8,000 yuan	134 (33.33)	Whether HCV antibody positive	
≥8,000 yuan	27 (6.72)	No	376 (93.53)
Nationality		Yes	26 (6.47)
Dai people	168 (41.79)	ATS use	
Han nationality	83 (20.65)	Not using ATS	57 (14.18)
Other	151 (37.56)	Using ATS	345 (85.82)
Gender		Whether to drink	
Female	6 (1.49)	No	34 (8.46)
Male	396 (98.51)	Yes	368 (91.54)
Anti-HCV positivity		Whether smoking	
No	376 (93.53)	No	13 (3.23)
Yes	26 (6.47)	Yes	389 (96.77)

### Demographic characteristics and anti-HCV positivity among ATS drug users

In the Table 2, among the 345 patients who used ATS drugs (meth tablets, crystal meth crystals or ecstasy), the average age was (33.2 ± 9.70) years old, and 135 patients were combined with other drugs, accounting for 39.13%. The people who used ATS drugs were mainly concentrated in 26–45 years old (66.09%), male (98.84%), primary school and below (48.99%), unmarried/separated/divorced/widowed (73.04%), dai nationality (42.32%), and the number of sexual partners was mainly concentrated in nine (IQR 3–10.76).

**Table 2:** Demographic and economic characteristics by anti-HCV positivity among ATS users (*N* = 345).

**Variable**	***N*(%)/*M*(IQR)**	**Anti-HCV positivity (%)/*M*(IQR)**	**Anti-HCV negativity (%)/*M*(IQR)**	***P***
Age group				0.032
≤ 25	74 (21.45)	4 (18.18)	70 (21.67)	
26–45	228 (66.09)	11 (50)	217 (67.18)	
≥46	43 (12.46)	7 (31.82)	36 (11.15)	
Gender				0.022
Female	4 (1.16)	2 (9.09)	2 (0.62)	
Male	341 (98.84)	20 (90.91)	321 (99.38)	
Education level				0.011
Primary school and below	169 (48.99)	6 (27.27)	163 (50.46)	
Junior high school	132 (38.26)	9 (40.91)	123 (38.08)	
High school and above	44 (12.75)	7 (31.82)	37 (11.46)	
Marital status				0.304
Unmarried/separated/divorced/widowed	252 (73.04)	14 (63.64)	238 (73.68)	
Married/Unmarried cohabitation	93 (26.96)	8 (36.36)	85 (26.32)	
Average monthly income				0.257
< 4,000 yuan	206 (59.71)	10 (45.45)	196 (60.68)	
4,000–8,000 yuan	117 (33.91)	11 (50.00)	106 (32.82)	
≥8,000 yuan	22 (6.38)	1 (4.55)	21 (6.50)	
Nationality				0.379
Dai people	146 (42.32)	9 (40.91)	137 (42.42)	
Han nationality	72 (20.87)	7 (31.82)	65 (20.12)	
Other	127 (36.81)	6 (27.27)	121 (37.46)	

In the population using ATS, 22 anti-HCV positivity cases were detected, with a positivity rate of 6.38% (22/345). Univariate analysis of the characteristics of ATS users found that there were differences in age group, gender, education level, co-use of other drugs, injection drugs with anti-HCV positivity (*P* < 0.05), while in the analysis of marital status, month income, ethnicity, number of sexual partners, age of first drug use, whether to drink or smoking, and whether to use condoms, there was no significant difference with anti-HCV positive testing (*P* > 0.05), as shown in Tables 2, 3.

**Table 3:** The relationship between risk-related behavior and anti-HCV positivity among ATS users (*N* = 345).

**Variable**	***N*(%)/*M*(IQR)**	**Anti-HCV positivity (%)/*M*(IQR)**	**Anti-HCV negativity (%)/*M*(IQR)**	***P***
Age of first drug use	20 (17-25)	22.5 (18-35)	20 (17-5)	0.070
Age of first sex	18 (16-19)	18 (17-20)	18 (16-19)	0.490
Whether to drink				1.000
No	27 (7.83)	1 (4.55)	26 (8.05)	
Yes	318 (92.17)	21 (95.45)	297 (91.95)	
Whether smoking				0.521
No	11 (3.19)	1 (4.55)	10 (3.10)	
Yes	334 (96.81)	21 (95.45)	313 (96.90)	
Whether condoms are used (spouse/constant sexual partner)				0.611
Never used	113 (32.75)	9 (40.91)	104 (32.20)	
Not always used	75 (21.74)	4 (18.18)	71 (21.98)	
Used every time	46 (13.33)	1 (4.55)	45 (13.93)	
Declined to answer	111 (32.17)	8 (36.36)	103 (31.89)	
Whether condoms are used (temporary partners)				0.142
Never used	45 (13.04)	0 (0)	45 (13.93)	
Not always used	33 (9.57)	1 (4.55)	32 (9.91)	
Used every time	57 (16.52)	3 (13.64)	54 (16.72)	
Declined to answer	210 (60.87)	18 (81.82)	192 (59.44)	
Number of sexual partners	9 (3–10.76)	7.5 (3.75–10.19)	9 (3–10.76)	0.488
Concomitant use of other drugs				< 0.001
No	210 (60.87)	5 (22.73)	205 (63.47)	
Yes	135 (39.13)	17 (77.27)	120 (36.53)	
Whether injecting drugs				< 0.001
No	289 (83.77)	11 (50.00)	280 (86.07)	
Yes	56 (16.23)	11 (50.00)	45 (13.93)	

### Logistic regression analysis of anti-HCV positive cases in ATS drug dependent patients

All socioeconomic factors were included in multivariate logistic regression analysis. The results showed that, compared with those who did not use other drug types (heroin, marijuana, *K* powder, etc.), the combined use of other types of drugs while using ATS increased the risk of anti-HCV positive testing (*OR* = 7.29, *95%CI*: 1.982–26.81, *P* = 0.003); Compared with the non-injection group, people who injected drugs have a higher risk of anti-HCV positive testing (*OR* = 6.823, *95%CI*: 1.898–24.525, *P* = 0.003). At the same time, the study found that ATS users with high school or above had higher anti-HCV positivity rates than those with primary school or below (*OR* = 5.718, *95%CI*: 1.172–27.908, *P* = 0.031). Compared with the ATS population with a monthly income of < 4,000 yuan, those with a monthly income of 4,000–8,000 are more likely to have anti-HCV positivity rate (*OR* = 4.825, *95%CI*: 1.325–17.566, *P* = 0.017). There is no significant difference in age, gender and number of sexual partners etc. (*P* > 0.05) as shown in Table 4.

**Table 4:** Multivariate logistic regression analysis of risk factors for anti-HCV positive cases.

**Variable**		***B***	***SE***	***Wald***	***P***	***OR(95%CI)***
Age		−0.098	0.068	2.105	0.147	0.906 (0.794–1.035)
Age of first drug use		0.054	0.042	1.683	0.195	1.056 (0.973–1.146)
Age of first sex		0.000	0.044	0.000	0.999	1.000 (0.918–1.089)
Number of sexual partners		0.006	0.021	0.094	0.760	1.007 (0.965–1.049)
Gender	Female					1.000
	Male	−1.241	1.616	0.59	0.443	0.289 (0.012–6.862)
Average monthly income	< 4,000 yuan					1.000
	4,000–8,000 yuan	1.574	0.659	5.699	0.017*	4.825 (1.325–17.566)
	≥8,000 yuan	0.683	1.437	0.226	0.635	1.979 (0.118–33.052)
Whether injecting drugs	No					1.000
	Yes	1.92	0.653	8.655	0.003*	6.823 (1.898–24.525)
Age group	≤ 25					1.000
	26–45	0.4	0.93	0.186	0.667	1.493 (0.241–9.235)
	≥46	2.448	1.888	1.682	0.195	11.569 (0.286–468.003)
Education level	Primary school and below					1.000
	Junior high school	−0.035	0.709	0.002	0.961	0.966 (0.241–3.874)
	High school and above	1.744	0.809	4.648	0.031*	5.718 (1.172–27.908)
Marital status	Unmarried/separated/divorced/widowed					1.000
	Unmarried cohabitation/married	0.113	0.645	0.031	0.861	1.12 (0.316–3.964)
Nationality	Dai people			0.382	0.826	1.000
	Han nationality	−0.291	0.716	0.165	0.685	0.748 (0.184–3.043)
	Other	−0.405	0.684	0.351	0.554	0.667 (0.175–2.548)
Whether to drink	No					1.000
	Yes	0.716	1.192	0.361	0.548	2.046 (0.198–21.152)
Whether smoking	No					1.000
	Yes	−0.825	1.354	0.372	0.542	0.438 (0.031–6.221)
Whether condoms are used (spouse/constant sexual partner)	Never used					1.000
	Not always used	−0.722	0.823	0.769	0.380	0.486 (0.097–2.439)
	Used every time	−1.972	1.254	2.473	0.116	0.139 (0.012–1.625)
	Declined to answer	−0.819	0.725	1.275	0.259	0.441 (0.106–1.827)
Whether condoms are used (temporary partners)	Never used					1.000
	Not always used	16.809	5,374.369	0	0.998	/
	Used every time	18.733	5,374.369	0	0.997	/
	Declined to answer	18.929	5,374.369	0	0.997	/
Concomitant use of other drugs	No					1.000
	Yes	1.987	0.664	8.939	0.003*	7.29 (1.982–26.81)

*Means *P* < 0.05.

## Discussion

To our knowledge, blood-borne disease infection is easy to occur among drug users. When amphetamine-type stimulants are used, the probability of high-risk behaviors increases, which increases the risk of blood-borne disease infection. Our results suggested that, a total of 402 drug users were investigated, and 85.82% (345/402) of the respondents used ATS. The anti-HCV positivity rates in the ATS population was 6.38% (22/345), while in the overall population of this study, the HCV infection rate was 6.47% (26/402), both of which were higher than the general population rate (3.2%) (14).

The younger age (33.2 ± 9.70) among ATS users suggested the group may be at earlier stages of drug dependent. Previous studies (15) found that women, poverty and injecting drug use were associated with anti-HCV positivity testing in the population using methamphetamine. In our study, 135 participants (39.13%) had a history of multiple drug use, including heroin, tramadol, cocaine and other opiate preparations. The combined use of other drug types (e.g., heroin, cannabis, and cocaine, and ketamine powder) increased the risk of HCV infection in this population (*OR* = 7.29, *95%CI*: 1.982–26.81, *P* = 0.003), which was consistent with the findings of Cai et al. (15) and Bao et al. (16). It may be because the combined use of drugs increased the probability and frequency of drug injection, thereby increasing the exposure to blood-borne viruses (7, 17). However, infection risk associated with ATS injection may differ depending on whether use ATS primarily or inject ATS in combination with addictive opiates such as herion, which was not focused on in this study regrettably.

Our study found, in the population using ATS drugs, the investigation on whether to use injection drugs also proved that the use of injection drugs was more likely to increase the risk of HCV infection (*OR* = 6.823, *95%CI*: 1.898–24.525, *P* = 0.003). This suggested that there was an urgent need to take appropriate measures for this population to address factors that may increase the risk of HCV infection. Several studies (15, 18) had found that people with lower literacy levels in ATS users were more likely to be exposed to drugs, thereby increasing the risk of HCV infection, but this study showed the opposite result. Compared with ATS users in primary school and below, ATS users with high school and above educational level were more likely to have the risk of HCV infection (*OR* = 5.718, *95%CI*: 1.172–27.908, *P* = 0.031), which might be because the respondents might have concealed information, leading to estimation errors. In addition, of the ATS population in this study, the number of sexual partners was mainly concentrated in nine (IQR3-10.76), which was related to the excitability of ATS, and ATS could enhance human sexual impulse, which might affect the risk of HCV infection. However, ATS users in our study reported no relationship with multiple sex partners (*P* = 0.76) contrasted with one study findings (19) where positive associations between ATS injection and multiple sex partners. Our study found that compared with a monthly income of < 4,000 yuan, new drug abusers with a monthly income of 4,000–8,000 yuan were more likely to develop HCV infection (*OR* = 4.825, *95%CI*: 1.325–17.566, *P* = 0.017). In general, the actual income may affect the anti-HCV positivity rate of ATS population, because the cost of new drugs is more, and the amount of funds may affect the frequency and mode of drug use.

A vitro experimental study (20) found that methamphetamine can destroy the innate immunity against HCV infection mediated by interferon alpha, and may play a role as a cofactor in the immune pathogenesis of HCV disease. At present, there is no effective vaccine to prevent HCV infection, mainly relying on popularizing relevant knowledge to people, focusing on high-risk groups, cutting off the transmission route, especially reducing the number of people who injected drugs and other drug users (21). Since 2014, the emergence of direct acting antiviral drugs has greatly improved the efficacy and safety of HCV treatment. It can achieve sustained virological response rate of more than 90%, and shorten the treatment time to 8–12 weeks (22, 23). The antiviral treatment of patients with chronic HCV infection has gradually entered the era of pan-genotype, and the pan-genotype direct acting antiviral drugs program is an effective means to eliminate viral hepatitis by 2030 (24, 25). When chronic HCV infection is diagnosed, antiviral drugs should be carried out as soon as possible, in order to cure the infection, and prevent cirrhosis, liver cancer, and other associated complications of the disease.

There are also some deficiencies in this study. First of all, most of the recruited people came from detention centers. ATS users are restricted. Although their fears are minimized during the investigation process, they may be worried about the information they provide, which increases the risk of information deviation. Secondly, small sample size and geographical location coverage may limit the detection of anti-HCV positive cases and potential risk factors in ATS users.

## Data availability statement

The raw data supporting the conclusions of this article will be made available by the authors, without undue reservation.

## Ethics statement

The studies involving human participants were reviewed and approved by Medical Ethics Committee of Kunming Medical University. The patients/participants provided their written informed consent to participate in this study.

## Author contributions

JinL and JY contributed to conception and design of the study and wrote sections of the manuscript. ML, JiaL, YZhao, RL, and YZhan conducts field research and data collection. ML performed the statistical analysis and wrote the first draft of the manuscript. All authors contributed to manuscript revision, read, and approved the submitted version.

## Funding

The survey was supported by National Natural Science Foundation of China (72274086, 81760617, and 81760111), NHC Key Laboratory of Drug Addition Medicine (2020DAMARA-003), Yunnan Province Science and Technology and Academic Leader Reserve Talents (202205AC160064), and Yunnan Province Innovation Team Cultivation Project (202005AE160002).

## Conflict of interest

The authors declare that the research was conducted in the absence of any commercial or financial relationships that could be construed as a potential conflict of interest.

## Publisher's note

All claims expressed in this article are solely those of the authors and do not necessarily represent those of their affiliated organizations, or those of the publisher, the editors and the reviewers. Any product that may be evaluated in this article, or claim that may be made by its manufacturer, is not guaranteed or endorsed by the publisher.
